# Impacts of childhood trauma on postpartum depression: prospective longitudinal study

**DOI:** 10.1192/j.eurpsy.2025.472

**Published:** 2025-08-26

**Authors:** E. B. Camargo Júnior, G. G. Silva, M. N. D. F. Fernandes, E. C. D. S. Gherardi-Donato

**Affiliations:** 1 University of Rio Verde, Rio Verde; 2 Federal University of Maranhão, Imperatriz; 3 Ribeirão Preto College of Nursing, Ribeirão Preto, University of São Paulo, Ribeirão Preto, Brazil

## Abstract

**Introduction:**

Women who experience childhood trauma may be at a greater risk of developing postpartum depression (PPD), which can result in significant harm to both mothers and their children. Few studies have longitudinally evaluated the effect of childhood trauma on PPD.

**Objectives:**

This study aimed to evaluate the impact of childhood trauma on PPD among Brazilian postpartum women.

**Methods:**

This prospective longitudinal study was conducted with 153 women evaluated at two time points: T1 (immediate postpartum) and T2 (three months postpartum). PPD symptoms were assessed using the Edinburgh Postnatal Depression Scale (EPDS) and childhood trauma was assessed using the Childhood Trauma Questionnaire (CTQ). To verify the differences in PPD scores in the periods assessed and in relation to childhood trauma, generalized estimating equations (GEE) were used. EPDS scores were categorized with values ≥10 defined as the presence of PPD. Multinomial logistic regression analyses were performed to evaluate the influence of trauma on PPD risk subgroups as follows: early PPD (risk of depression at T1), late PPD (risk of depression at T2), and chronic PPD (risk of depression at T1 and T2).

**Results:**

The results demonstrated that women who suffered trauma in childhood had significantly higher EPDS scores at both time points evaluated when compared to women who did not suffer from trauma. However, there was no significant difference in EPDS scores over time or in the interaction between time and childhood trauma, indicating that PPD scores and the impact of childhood trauma on PPD remained constant over time. All types of childhood trauma were significantly associated with late or chronic PPD. Emotional abuse, physical abuse, and emotional neglect are significantly associated with early PPD.

**Conclusions:**

The present study demonstrated that women who experienced childhood trauma had significantly greater symptoms of PPD. However, PPD symptoms did not vary between the two assessments and remained stable. Mental health screening and interventions must be adopted during pregnancy monitoring and in the postpartum period.

**Disclosure of Interest:**

None Declared

